# The role of sleep in neuromuscular disorders

**DOI:** 10.3389/fneur.2023.1195302

**Published:** 2023-06-29

**Authors:** Corrado I. Angelini, Carl Ansevin, Gabriele Siciliano

**Affiliations:** ^1^Department of Neurosciences, University of Padua, Padua, Italy; ^2^Department of Neurosciences, Sleep Center of the Ohio Neurologic Institute, Youngstown, OH, United States; ^3^Department of Neurosciences, University of Pisa, Pisa, Italy

**Keywords:** sleep, neuromuscular disorders, Duchenne, limb-girdle dystrophy, ataxias

## Abstract

Sleep represents a major frontier both in clinical myology and as a new possibility for delivering treatment to neuromuscular patients since various neuromuscular cases present a variable degree of disordered sleep and such conditions should be diagnosed and prevented, i.e., sleep apnea and hypoxemia. These sleep disorders are present in dystrophinopathies and in various types of limb-girdle muscular dystrophies (LGMD). Excessive daytime sleepiness (EDS) is found in patients affected by spastic paraparesis or cerebellar ataxia but is rather common in both myotonic dystrophy type 1 and 2, and the correction of sleep disorders is therefore important to improve their daily quality of life (QoL) and consequent daily functioning. Other types of sleep dysfunction such as insomnia, a reduction in rapid eye movement (REM) sleep, loss of normal REM, or sleep-disordered breathing are found in other disorders including myasthenia, ataxias, spastic paraparesis, Charcot–Marie–Tooth disease, and neurogenic disorders, including polyneuropathies, and need appropriate treatment. Research done on this topic aims to incorporate a variety of nuances in metabolic disorders such as those in late-onset Pompe disease and are such as those in late-onset Pompe disease who are susceptible to enzyme replacement therapy (ERT). The overarching goal is to explore both the diagnosis and methodology of sleep-related problems in both genetic and acquired neuromuscular disorders. We also review the type of available treatment opportunities utilized to improve neuromuscular patients’ QoL.

## Introduction

The present review aims to introduce how to diagnose sleep-related problems in a series of both genetic and acquired neuromuscular disorders and examine the available treatment opportunities that might be utilized in clinical practice to improve neuromuscular patients’ quality of life (QoL).

There are disorders such as cerebellar ataxia or spastic paraparesis where, after correction of sleep disorders, there is a recognizable improvement of cognition. This cognitive feature applies to severe cases of limb-girdle muscular dystrophies (LGMD) where nocturnal bilevel positive airway pressure (BiPAP) treatment brings individuals back to their usual daily life activity and possibly to the work environment.

Patients with myasthenia gravis (MG) and other neuromuscular disorders are sometimes difficult to treat because of their respiratory state can be precarious. This can be exacerbated with medications (e.g. benzodiazepines), infections, etc. and they can be thrown into myasthenic crises and respiratory failure. A decades old axiom for the treatment of patients with MG has long been “When in a myasthenic crisis first secure the airway!” With the advances in sleep medicine and treatment of nocturnal sleep apneas disorders, it is no longer necessary to wait for a myasthenic crisis or respiratory failure to start to protect the airway. If sleep disordered breathing (sleep apneas, hypopneas, etc) are identified and treated early, valuable time can be saved and perhaps costly hospitalizations avoided. This is particularly important in the field of myasthenia gravis and neuromuscular junction (NMJ) disorders where available treatments may take time before they become effective. This includes long recognized effective treatments (steroids and immunosuppressives) and several new drugs (compliment inhibitors and anti-FcRn therapy) in patients with steroid-resistant MG. It is well-recognized that with the use of biPAP and other modalities of NIV for sleep disordered breathing in these patients, it may be possible not only to avoid myasthenic crises, but also to improve patients’ quality of life. In patients with neuromuscular disorders, including myasthenia and its various forms (e.g. DOK7, MUSK, etc), the use of biPAP may result in improvement in QoL and cognition.

Clinical and drug treatment of excessive daytime sleepiness (EDS) in patients with cerebellar ataxia, spastic paraparesis, myotonic dystrophy type 1 (1–6), and several LGMD cases represents a major advancement in the field of patients’ daily management, as it may improve their QoL perception and therefore improve both their independence and self-satisfaction.

To experienced clinicians in the myology field, the observation of latent depression in many NMD patients is common; what is important to acknowledge is that there is a correlation between such depressive symptoms and sleep quality. Furthermore, it is necessary to consider that disrupted sleep induces negative effect both due to frequent episodes of sleep apnea and consequent hypoxia in muscle and NMJ disorders.

## Neuromuscular diseases

Sleep is an approachable frontier in clinical myology to improve the treatment of neuromuscular diseases; many neuromuscular patients present various degrees of disordered sleep. We here propose a schematic table (Table 1) of various disease entities and their frequency and an algorithm to diagnose and prevent both sleep apnea and related hypoxemia.

**Table 1:** Neuromuscular disorders that could affect sleep and their possible treatments.

Clinical entity	Type of sleep dysfunction	Severity/treatment
Myasthenia gravis	Insomnia, arousals	IVIG
Ataxias	Insomnia	Tranquilizers

FSHD	Respiratory insufficiency	BiPAP
Duchenne MD	Sleep apnea	BiPAP
Myotonic dystrophy	Excessive daytime sleepiness	Modafinil, CBT
Sarcoglycanopathy (LGMDR3-5)	Sleep apnea	BiPAP
LGMD R1	Respiratory insufficiency	BiPAP
LGMD R2	Sleep apnea	BiPAP

LOPD	Nocturnal ventilation	ERT, exercise, nutrition
Spastic paraplegia	Insomnia, spasms	Muscle relaxants
Diabetic polyneuropathy	Cramps, pain	Glucose control
Myasthenic syndromes	SDB	BiPAP, salbutamol treatment

SCA	Insomnia	Tranquilizers
Charcot–Marie–Tooth carpal tunnel syndrome	Cramps Cramps, paresthesia	Gabapentin surgery

The arousal system and brain central nervous system (CNS) are variably deranged in congenital, childhood, juvenile, and adult Myotonic Dysitrophy type 1 (DM1) and, to a lesser extent in Myotonic Dystrophy type 2 (DM2), where a congenital form has not been so far recognized. The various forms of myotonic dystrophy type 1 drugs, such as modafinil, and cognitive behavioral treatment (CBT) is used and appear useful to prevent EDS. Mexiletine at the dosages of 150 to 200 mg Three times a day (TID) (7) has been proven to be effective in reducing myotonia with no significant effect on cardiac conduction over a 7 weeks treatment period, except for possible first-degree Atrio-ventricular (AV) Block (8). CNS stimulants such as modafinil can be used for excessive daytime sleepiness (9). For those with high risk of cardiac arrhythmias (10), a yearly cardiological referral is strongly recommended to evaluate the need for an intra-cardiac defibrillator device insertion. Sleep and respiratory studies with gas analysis should be done to evaluate the need for non-invasive nocturnal ventilation.

Physical therapy can be performed to improve muscle function; some studies evidenced that exercise programs and functional electric stimulation can be performed safely and result in a temporary improvement of patients’ muscle function (11–13). A possible limiting factor in the development of the applicability of CBT and exercise guidelines is represented by the lack of uniformity in the proposals of exercise protocols and outcome measures and this needs further refinement and guidelines (14). In the field of myotonic disorders, physiotherapy may also help with the treatment of respiratory failure, dysarthria, and dysphagia. In the field of neuromuscular disorders with CNS involvement, the most common changes found in DM1 are characteristic white matter lesions in temporal lobes, of possibly developmental origin. Also, in mitochondrial encephalomyopathies, sleep-related disturbances might be present in 60% of cases and EDS in 30%, especially in cases with psychiatric disorders, brain imaging might be unspecific but in some cases, there is leukodystrophy, gray matter atrophy, or hyperintensities in the putamina, with involvement of the caudate nuclei, thalami, and brain stem, where sleep centers are localized.

A very common muscular dystrophy with large clinical variability is represented by facioscapulohumeral dystrophy, now subdivided into FSHD1 and FSHD2, with a spectrum of clinical presentations from infantile to adult oligosymptomatic patents. In some childhood cases of FSHD1, respiratory muscle weakness is prominent with severe clinical manifestations such as pectus excavatum and scoliosis and might be associated with sleep-disordered breathing (SDB), which produces obstructive sleep apnea (OSA) and is associated with hypoxemia for nocturnal hypoventilation (NH). Their prevalence is still to be evaluated in large series since data are relatively scarce.

By investigation with questionnaires (15), both adult FSHD and BMD patients (Becker muscular dystrophy or mild dystrophinopathy) reported worse sleep than controls. In particular, it has been observed that FSHD patients scored significantly worse than the controls on the Insomnia Severity Index.

## Risk factors

In limb-girdle muscular dystrophy (LGMD) syndromes, there are several patients, especially in the more severe recessive LGMD forms (LGMD R), suffering from respiratory disorders, mostly in cases with a juvenile onset. The most frequent recessive forms are represented by LGMD R1 or calpainopathy and LGMD R2 or dysferlinopathy, and in developing countries they are represented mostly by sarcoglycanopathy patients (LGMD R3-R5) who suffer from OSA and SDB, which might be treated by Continuous positive airway pressure (CPAP). Similar episodes of variable extents have been observed in late-onset Pompe disease (LOPD) patients, who often need a ventilator, and when they are treated with ERT, they usually present decreased hours on the ventilator and thus sleep in these adult Pompe patients is improved.

It is widely known that, in adolescent boys with Duchenne muscular dystrophy, there is poor sleep quality and nocturnal ventilation is a necessary treatment of their OSA. However, clinical follow-up and observations of sleep quality in milder forms of dystrophinopathy such as BMD, where muscle weakness, cramps, and fatigue with exercise limitation and myoglobinuria occur, and evaluation of consequent QoL, pain, and fatigue remains limited.

Recent studies have shown that sleep treatments for SDB improve also QoL in a series of NMD patients and promotes better daily function, but several problems have still to be evaluated.

We still do not know which patients with neuromuscular disease are at higher risk of developing a sleep dysfunction and therefore should be actively monitored.

Several neurological associations and neuromuscular networks have produced guidelines on the impact of sleep disorders and their treatment. These guidelines describe in most except the mildest forms of neuromuscular diseases high or moderately high the risk of a severe course.

The clinical features associated with a high risk of severe SDB include, for instance, the presence of prominent chest muscular weakness or diaphragmatic weakness, resulting in a forced vital capacity (FVC) of less than 60% predicted; such a low FVC is observed especially in patients with kyphoscoliosis but also in rigid spine cases common in LOPD or laminopathies.

Another risk factor that might be observed in NMJ or motor neuron disease patients is the use of ventilation *via* a mask or tracheotomy. In NMJ cases, it is common to find a weak cough and weak airway clearance due to oropharyngeal weakness. In both infantile and adult spinal muscular atrophy, patients often present with the presence of tracheostomy.

LGMD patients and dystrophinopathy cases might present cardiac involvement, and the use of medication for heart involvement might worsen during sleep.

In metabolic myopathies, one should monitor the risk of deterioration with fasting or infection, since they might present a sudden deterioration overnight for muscle atonia during REM sleep. Another prominent risk is rhabdomyolysis occurring with fever and fasting. In general, in all NMD diseases, severity and poor quality of sleep is due to the occurrence of concomitant diabetes and obesity.

In identifying possible risk factors, the clinician should analyze the individual characteristics of neuromuscular patients, and evaluate pharyngeal weakness and the presence of macroglossia. It is important to remember that bulbar manifestations, dysphagia, and aspiration phenomena are linked especially in motor neuron disorders. Factors such as low FVC, diaphragmatic weakness, and consequent defective lung volumes predispose patients to the development of obstructive events.

Another risk factor resulting from bulbar center malfunction is central sleep-disordered breathing that might occur in association with cardiomyopathy in muscular dystrophies, DM1, or LOPD, and has been attributed to instability in the control of breathing. This also represents a major contribution to diaphragmatic weakness. An opposite action such as the recruitment of accessory respiratory muscles might mitigate diaphragm weakness.

A polysomnography might be required to evaluate the reduction in REM sleep, and loss of nomal atnia during REM in indivdual LOPD or DM1 patients, which in single cases might have a paradoxical beneficial effect and protect against SDB.

On this basis, several risk factors appear with increasing age in several NMDs and are at difference with the normal individual where sleep is a normal restorative function; in NMD patients, if sleep disorders are untreated, a definite cause of disease course deterioration can occur, with worsening of patients’ quality of life.

In all but the mildest forms of NMD, airway manifestations can take weeks to months or even years to evolve, but respiratory follow-up is important for clinical myologists, who should recognize based on direct investigation and laboratory exams two major respiratory phenotypes, characterized by rapid or slow progression to respiratory failure.

We support the clinical observation that both type and age of the onset of airway problems have to be accurately monitored by a clinical myologist, as well as their evolution over time. This type of care will support practicing myologists in the diagnostic assessment, since, in general, the precise knowledge of the characteristics of NMD patients’ respiratory phenotypes will be useful and possibly increase the level of collaboration with other allied professionals such as dieticians, physical therapists, occupational therapists, geneticists, pulmonologists, and chest therapists, resulting in the increased level of support in the common task of the multidisciplinary management of such patients (16).

## Diagnostic clues

To make a diagnosis, the physician will use the patient’s medical history, sleep history, and physical exams. The symptoms suggestive of desaturation during night hours are headache and loss of vigilance during the day.

The treating physician might suggest a sleep polysomnography, which can be done in a hospital center, but nowadays also at home. The most common type of polysomnography monitors and registers data about body functions, such as movements, respiration, and blood pressure during sleep. In addition, a polysomnography records Electroencephaloraphy (EEG) brain waves, the oxygen saturation through pulse oximetry, and registers the heart rate using Electrocardiography (EKG) monitoring and breathing during sleep. It also measures eye movements to detect REM sleep and concomitant leg movements or atonia. An Electromyography (EMG) is concomitantly done and video monitoring might be useful.

Depending on the available facilities in various countries, this type of sleep study may be done in a sleep disorders unit within a hospital or at a sleep center. A polysomnography will be usually performed at night, but it may be done during the daytime for some NMD patient who usually sleep during the day. Other important clues are given by pulse oximetry, spirometry, and blood gas analysis.

The relevance of such a test is not only diagnostic since polysomnography might help determine a treatment plan when the neuromuscular patient has been diagnosed with a sleep disorder.

**Figure 1:**
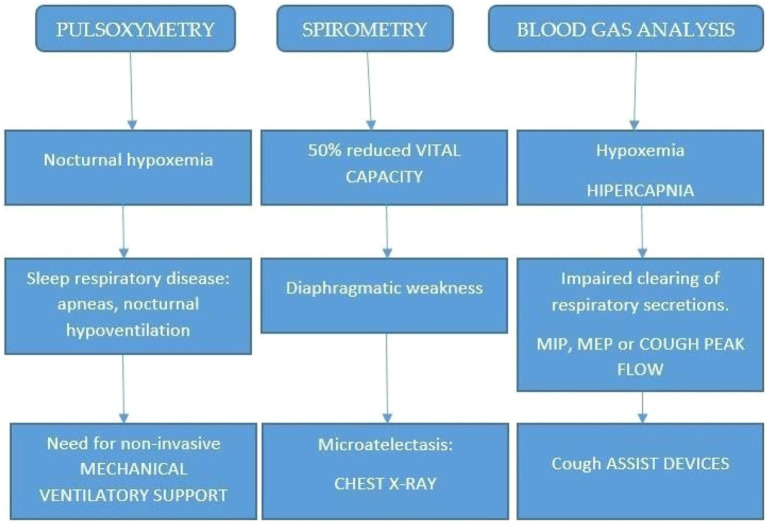
Proposed algorithm for diagnosing sleep disturbances in neuromuscular patients.

In addition, a polysomnography might be used to adjust the patient to the home environment and use nocturnal ventilation. In Figure 1, an algorithm is reported for the diagnosis and management of NMD patients. The collection of main symptoms, such as morning headache, dyspnea, and Hoover sign (forced inspiration), directly or by a questionnaire, as an initial step, will be extremely useful.

## Scope and treatment

Sleep disorders are a common problem in neuromuscular disorders and usually associated with a poor quality of life, worsening of muscle weakness, and systemic implications. In the literature, there are still few articles exploring the topic of sleep problems in neuromuscular disorders; the most recent relevant papers are on DM1 and DM2 (17, 18), and a focus on other neuromuscular disorders is needed. There is still a lack of an extensive review of the literature about the prevalence of sleep disorders in neuromuscular diseases, and only further research will allow the collection of relevant epidemiological data.

We propose a review of sleep disorders in neuromuscular disorders since this is a hot topic and it is important to further construct new knowledge about this topic in the literature.

The pathophysiological mechanisms and pathological implications of sleep problems in neuromuscular disorders need more extensive coverage and both clinical and translational research in the field. This minireview focuses on a special tool available in the treatment of sleep disorders with special emphasis on the role and life-saving mechanism of ventilatory support. We aim to emphasize that although ventilatory support is important and can improve QoL and prolongs also lifespan, other treatments are available and, in other cases, drugs or other types of intervention appear useful.

In this review, we want to emphasize the importance of following sleep disorders in clinical myology practice: one should be aware that EDS is frequent in DM1 cases, but the concentration difficulties present in DM1 might be treated with variable benefit with modafinil (8). A milder sleep problem is usually observed in DM2 patients (18).

An important risk factor to be treated for is nocturnal apneas that occur both in Duchenne Muscular Dystrophy (DMD) and spinal muscular atrophies (SMA), where nocturnal ventilation can prolong the life span. One concomitant factor changing the scenario of these child disorders is represented by the beneficial effect of current therapy from steroids for DMD to personalized drugs (AON; Spinraza, etc.). Without intervention, many children with SMA type 1 die from respiratory failure before their second year of life. While assisted ventilation has improved survival, it often results in ventilator dependence. The development of new Suvival Motor Neuron (SMN) augmenting therapies has renewed optimism, but their long-term impact on respiratory functioning is uncertain, and non-invasive respiratory support remains an important part of SMA management. Despite the importance of respiratory support in SMA, knowledge of sleep disorders in this population is limited; for the high prevalence of nocturnal alveolar hypoventilation in SMA, overnight monitoring of gas exchange or a formal sleep study is recommended. Non-invasive ventilation (NIV) should be initiated for isolated nocturnal hypoventilation and managed by a multidisciplinary pediatric respiratory team with expertise in NIV.

In both such genetic motor neuron disorders and in the realm of muscular dystrophies, personalized medicine is advancing and proposing new treatments such as ataluren and tofersen; therefore, all these advances might extend both QoL and lifespan.

In the LGMD subtypes, i frequent to observe that most adult calpainpthy patients (LGMD R1) present prominent respiratory insufficiency and sleep apnea, simlar observations are possible in dysferlinopathy cases in advanced stages (LGMD R2) and present and common in most sarcoglycanopathy cases (LGMD R3-R5).

We have observed during ERT that in patients with late-onset Pompe disease (LOPD), sleep hours improve; in addition, concomitant exercise and nutrition appear useful to improve QoL (19). Sleep disturbances are common in carpal tunnel syndrome (CTS), a treatable and easily diagnosed disorder; its effects on quality of life and the cost that the CTS burden generates to society due to disordered sleep might be useful to remember in clinical practice (20). Amyloidosis is now a neurogenic disorder amenable to drug treatment and such neuropathy might present often as a sleep disorder.

Albeit rare, congenital myasthenic syndromes might present respiratory insufficiency. In particular, infants affected by Acetylcholinesterase collagenic tail peptide also known as AChE Q subunit (COLQ) or Rapsyn defects are at risk of briefly resolved unexplained events and sleep disorders of breathing that might benefit by using the BiPAP apparatus; similarly, in severe myasthenic syndromes, due to DOK7 and MUSK, there are remarkable improvements in cognition and quality of life in patients with these NMJ disorders (Table 1).

## Author contributions

CoA drafted the manuscript. CaA and GS revised the manuscript and contributed to individual sections. All authors contributed to the article and approved the submitted version.

## Conflict of interest

The authors declare that the research was conducted in the absence of any commercial or financial relationships that could be construed as a potential conflict of interest.

## Publisher’s note

All claims expressed in this article are solely those of the authors and do not necessarily represent those of their affiliated organizations, or those of the publisher, the editors and the reviewers. Any product that may be evaluated in this article, or claim that may be made by its manufacturer, is not guaranteed or endorsed by the publisher.
